# Whole-body low-dose CT can be of value in prostate cancer decision-making: a retrospective study on 601 patients

**DOI:** 10.1186/s13244-023-01475-w

**Published:** 2023-07-16

**Authors:** Mohammadreza Chavoshi, Seyed Ali Mirshahvalad, Sara Zamani, Amir Reza Radmard, Babak Fallahi, Seyed Asadollah Mousavi

**Affiliations:** 1 Department of Hematology-Oncology, Hematology-Oncology and BMT Research Center/Tehran University of Medical Sciences, Tehran, Iran; 2 Department of Radiology, Shariati Hospital, Tehran University of Medical Sciences, Tehran, Iran; 3 Joint Department of Medical Imaging (JDMI), University Medical Imaging Toronto (UMIT), University Health Network, Mount Sinai Hospital and Women’s College Hospital, University of Toronto, Toronto, ON Canada; 4 Department of Nuclear Medicine, Shariati Hospital, Tehran University of Medical Sciences, Tehran, Iran; 5 School of Medicine, Isfahan University of Medical Sciences, Isfahan, Iran

**Keywords:** Prostate, Computed tomography, Bone, Metastasis, ^68^Ga-PSMA

## Abstract

**Objectives:**

To evaluate the diagnostic value of whole-body low-dose computed tomography (CT) to detect bone metastasis in prostate cancer (PCa) patients and its possible utility in therapeutic decision-making. Also, to determine the valuable CT features for lesion characterisation.

**Methods:**

This IRB-approved retrospective study reviewed PCa patients who underwent ^68^Ga-PSMA PET/CT in our centre from March 2017 to August 2022. Two board-certified radiologists and one nuclear medicine specialist reported all whole-body low-dose CT scans separately, unaware of the ^68^Ga-PSMA-PET results. The per-lesion and per-patient diagnostic performances were calculated. Also, the significance of CT features was evaluated. Moreover, the inter-observer agreement was analysed. A two-tailed *p* value < 0.05 was considered significant.

**Results:**

From 727 reviewed PCa patients, 601 (mean age = 68.7 ± 8.1) were found to be eligible, including 211 (35.1%) referrals for initial staging and 390 (64.9%) for evaluating the extent of the disease after biochemical recurrence. Per-patient diagnostic analysis for three reviewers showed 81.0–89.4% sensitivity and 96.6–98.5% specificity in detecting osteo-metastasis. It was able to correctly detect high-burden disease based on both CHAARTED and LATITUDE criteria. Regarding the value of underlying CT features, size > 1 cm, ill-defined borders, presence of soft-tissue component, and cortical destruction were statistically in favour of metastasis. Also, Hu > 900 was in favour of benign entities with 93% specificity.

**Conclusions:**

Although not as accurate as ^68^Ga-PSMA PET/CT, whole-body low-dose CT might precisely classify PCa patients considering therapeutic decision-making. Additionally, we proposed diagnostic CT features that could help radiologists with better characterisation of the detected lesions.

**Critical relevance statement:**

The whole-body low-dose CT can be considered valuable in the clinical decision-making of prostate cancer patients. This modality may obviate performing multiple imaging sessions and high-cost scans in patients diagnosed with the high-burden disease.

**Graphical abstract:**

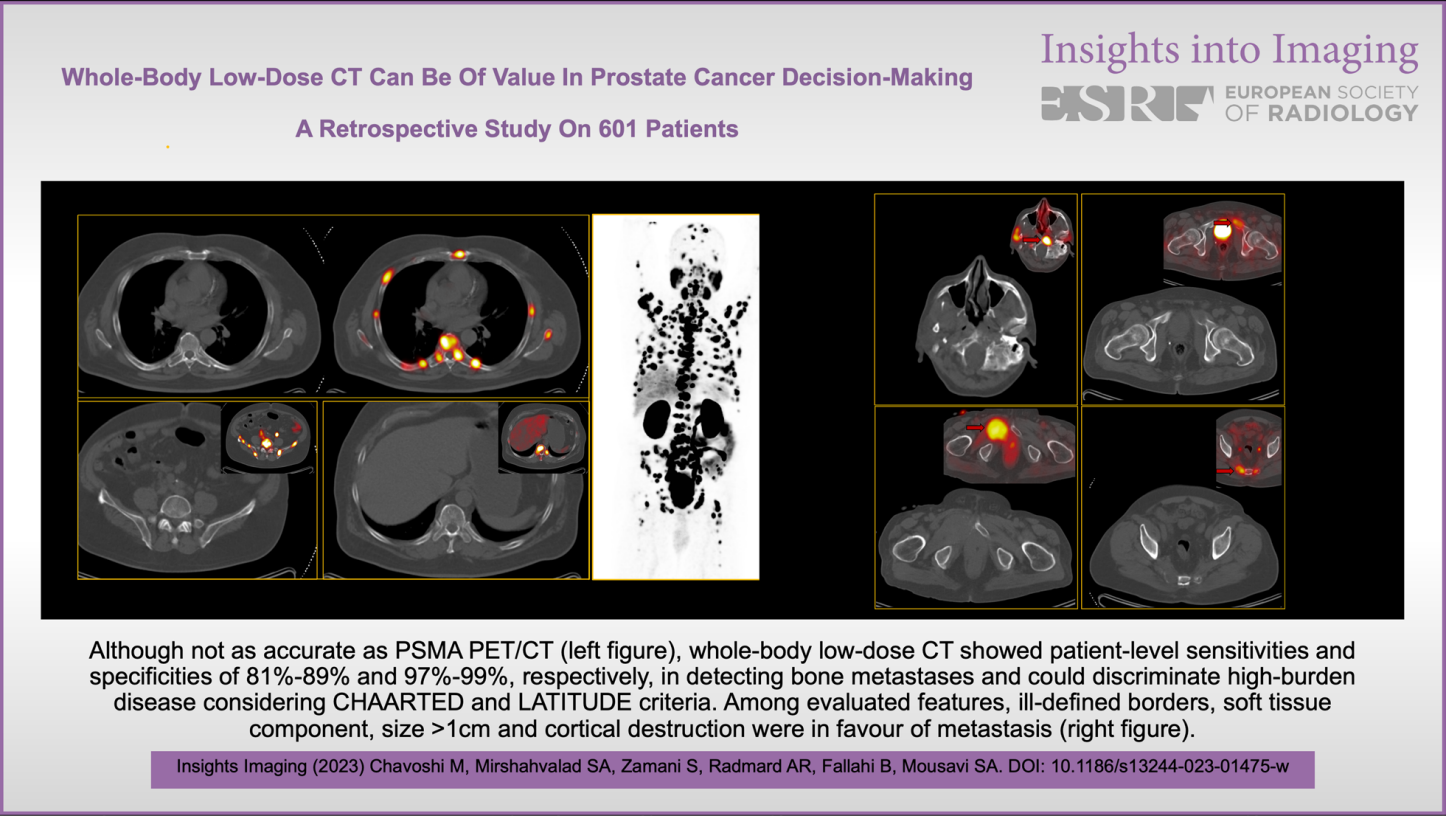

## Key points

1. The whole-body CT showed 81.0%-89.4% sensitivity and 96.6–98.5% specificity in detecting osteo-metastases.
2. The whole-body CT could detect high-burden disease considering CHAARTED and LATITUDE criteria.
3. Regarding CT features, lesions with Hu>900 were found benign with 93% specificity.

## Introduction

Bone is the second most common site of metastasis in prostate cancer (PCa) [1]. Identifying osteo-metastasis is highly crucial from both prognostic and therapeutic standpoints. Thus, it is included in different decision-making criteria in this field. The latest guidelines recommend evaluating bone metastasis in intermediate- and high-risk patients in the initial staging [2–4]. The CHAARTED trial defined the high-volume disease in initial staging as ≥ 4 osteo-metastases (≥ 1 beyond vertebrae and pelvis) or the presence of visceral metastases [3]. The LATITUDE trial defined high-risk patients as meeting ≥ 2 of three criteria: (1) Gleason score ≥ 8, (2) ≥ 3 osteo-metastases, and (3) measurable visceral metastasis [4]. Additionally, detecting osteo-metastasis leads to adding bone-modifying drugs to patient treatment [5].

Traditionally, bone radiography and bone scintigraphy (BS) were used for skeletal assessment. Although BS still is recommended in guidelines, more accurate modalities, such as ^68^Ga-PSMA positron emission tomography/computed tomography (PET/CT), have been developed [6, 7]. Particularly, in osteo-metastasis, ^68^Ga-PSMA PET/CT was highly accurate, showing 96–97% sensitivity and 99–100% specificity [8, 9]. Beyond its high accuracy, the interobserver agreement for this modality is also substantial, leading to a confident patient diagnosis [10]. However, the test is expensive and is not widely available in all medical centres, especially in developing countries.

CT is among the most well-known, available, and affordable imaging modalities worldwide. Due to the accessibility, this cross-sectional imaging modality is recommended for evaluating PCa metastasis [2, 11]. Clinicians mostly use this modality for evaluating chest or abdominopelvic lymph-node metastasis [12]. Previous studies have evaluated its accuracy in osteo-metastasis and have proposed limited diagnostic accuracy [8, 13]. Therefore, it is recommended that CT is accompanied by BS [2]. Given these concerns that limit reading confidence, radiologists are frequently reluctant to devote special attention to suspicious lesions on CT, unless confirmatory modalities corroborate the diagnosis.

Although osteo-metastasis detection is critical, determining the *exact* number/location of lesions is not of much importance. Thus, CT might be undervalued in bone assessment. Based on CHAARTED and LATITUDE criteria, all patients with high-volume/risk status receive the same treatment. Additionally, osteo-metastasis local therapy is only considered when clinical symptoms (e.g. pain) or a high risk of fracture exists [2, 14]. Therefore, practically, correct categorisation is the key to adopting the proper treatment in PCa patients.

Thus, we hypothesised that performing whole-body low-dose CT for staging or evaluation of biochemical recurrence (BCR) in PCa patients may be of value, classifying them with a less-expensive modality. The primary goal of this study was to determine the full scope of the whole-body low-dose CT diagnostic performance in detecting osteo-metastases and its utility in the decision-making for PCa patients. The secondary goal was to determine the CT features that could be used to differentiate benign and metastatic lesions.

## Materials and methods

**Fig. 1:**
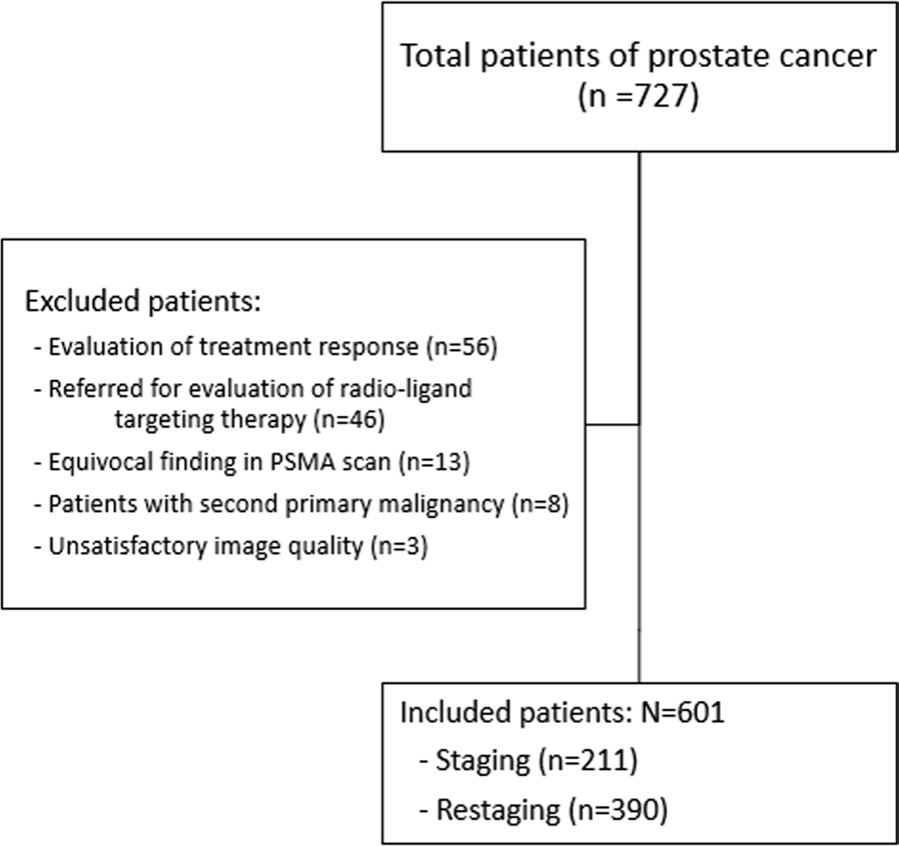
Flowchart of patients’ selection

This is a cross-sectional retrospective study conducted according to the guidelines of the Declaration of Helsinki and approved by the institutional review board and ethics committee of the university (IR.TUMS.HORCSCT.REC.1400.027). From March 2017 to August 2022, all PCa patients referred for ^68^Ga-PSMA PET/CT were included (Fig. 1). Patients with the following criteria were excluded from the study: (1) evaluation of response to treatment, (2) patients with second primary malignancy and (3) patients referred for the evaluation of radioligand targeting therapy. Due to the high sensitivity and specificity of the ^68^Ga-PSMA PET/CT scan for detecting PCa bone metastasis, we considered it as the reference standard.

### ^*68*^*Ga-PSMA PET/CT acquisition*

The ^68^Ga-PSMA was produced according to the standard protocols provided by the manufacturer. Patients were not subjected to any special preparation before imaging. Approximately 60 min after the injection of a weight-adjusted dose of 2–4 MBq/kg (170 ± 22 MBq), whole-body PET/CT (Biograph 6 True Point HD, Siemens Medical Solutions, Erlangen, Germany) was performed. Image acquisition was made from vertex to mid-thigh. The CT component features were 80 mAs, 120–130 keV, a pitch of 1.3, and a slice thickness of 5 mm. Immediately after, maintaining the patient's position, PET acquisition (4 min/bed position) was performed. PET images were reconstructed after CT-based attenuation correction using the ordered subset expectation maximisation (OSEM) algorithm (2 iterations, 21 subsets) and post-reconstruction smoothing with a Gaussian filter.

### Image interpretation

Two board-certified radiologists (one junior with 4 years and one senior with more than 10 years of experience in reading CT) and one senior nuclear medicine specialist reported all whole-body CT scans separately, unaware of the ^68^Ga-PSMA PET results. The three readers were blinded to each other’s reported findings. Also, all readers were blinded to patients’ demographics, clinical data, underlying conditions and referral indications. Reviewers were asked to report all suspicious lesions for each patient and decide whether the lesion was benign or metastatic. The image review began with a default bone window (centre 450/ width 1500 Hounsfield unit [Hu]) that could be adjusted at the reader’s discretion. To prevent a biased increase in true-negative results, degenerative changes, geode lesions, Schmorl's nodes, simple fractures and typical hemangiomas were not reported. Additionally, to prevent a biased increase in true positive findings, patients with > 10 metastatic lesions were reported as extensive. To reach a consensus on the reporting template and terminology, reviewers reported 20 patients unblinded to each other, who were excluded from the final analysis. The reported features were: location, lytic/sclerotic nature, presence of a fracture or soft tissue component, having an ill-defined border, causing cortical destruction, presence of asymmetric growth plane (0: lesion with similar diameters in all axes, 1: lesion with one dominant axis), its maximum transverse diameter (reported ≥ 1 cm or not) and mean Hu. The bone evaluation with hybrid ^68^Ga-PSMA PET/CT was done separately by the before-mentioned nuclear medicine specialist after finishing all CT-only reports (with a three-month interval to limit recall bias) along with another expert nuclear medicine specialist in consensus. The interpretation of the reference standard was made based on the ^68^Ga-PSMA avidity of the lesions, considering the known pitfalls of ^68^Ga-PSMA imaging (e.g. hemangioma, fracture, Paget’s disease). In case of high suspicion for false ^68^Ga-PSMA PET/CT results, if available, patients’ clinical records were considered for precise assessment. Uncertain or equivocal findings on ^68^Ga-PSMA PET/CT images (due to imaging artefacts, i.e. halo, motion, and equivocal PET/CT findings that could not be precisely interpreted after also reviewing patients’ history, additional imaging or follow-up scans) were excluded from the analyses.

### Statistical analysis

The per-lesion and per-patient diagnostic performances were calculated for each reviewer, including sensitivity, specificity, positive predictive value (PPV), negative predictive value (NPV) and accuracy. Patients with extensive (> 10) metastases or without any suspicious lesion were only included for per-patient analysis. Also, the diagnostic performance of each reviewer in the initial staging of the patients was evaluated based on the cut-off values provided in CHAARTED and LATITUDE criteria to determine how much the reports could potentially affect the patient’s management. Based on CHAARTED criteria, any patient who had ≥ 4 osteo-metastases with at least one extra-axial lesion was considered “high volume” [3]. In LATITUDE criteria, patients with ≥ 3 bone metastases were assigned as “high risk” [4]. To determine the diagnostic performance in BCR, we categorised the patients into non-metastatic, oligometastatic (1–3 lesions) and poly-metastatic (≥ 4 lesions) groups based on the therapeutic approach [15].

To assess the relation of each feature in the CT scan with the probability of malignancy, the Mann–Whitney *U* and Chi-square tests were conducted for continuous and categorical data, respectively. Also, the odds ratio of the significant features was calculated. To determine the proper cut-off for Hu, we used receiver operating characteristic (ROC) curve analysis and prioritised the specificity in case of equal summation (Youden index). For this issue, the average of Hu values measured by all reviewers was used. The benign lesions were entered in analyses if reported by at least two reviewers.

To find out the inter-observer variability among the three reviewers, Krippendorff's alpha coefficient was calculated. Also, a one-by-one agreement among every two reviewers was calculated by Cohen’s kappa in a listwise deletion manner. The results were interpreted as: 0.01–0.20 indicated none to a mild agreement, 0.21–0.40 indicated fair agreement, 0.41–0.60 indicated moderate agreement, 0.61–0.80 indicated good agreement and 0.81–1.00 indicated nearly perfect agreement.

All analyses were done by IBM SPSS Statistics (ver.25, IBM Corp., Armonk, N.Y., USA). A two-tailed *p* value < 0.05 was considered significant.

## Results

A total of 727 PCa patients were referred to our tertiary centre for ^68^Ga-PSMA PET/CT from March 2017 to August 2022. After applying the exclusion criteria, 601 patients (mean age = 68.7 ± 8.1) were found to be eligible to enter the study. Figure 1 shows the flowchart of the patient selection process. Regarding indication, 211/601 (35.1%) patients were referred for initial staging, and 390/601 (64.9%) patients were referred for the evaluation of the extent of the disease after being diagnosed with BCR.

**Fig. 2:**
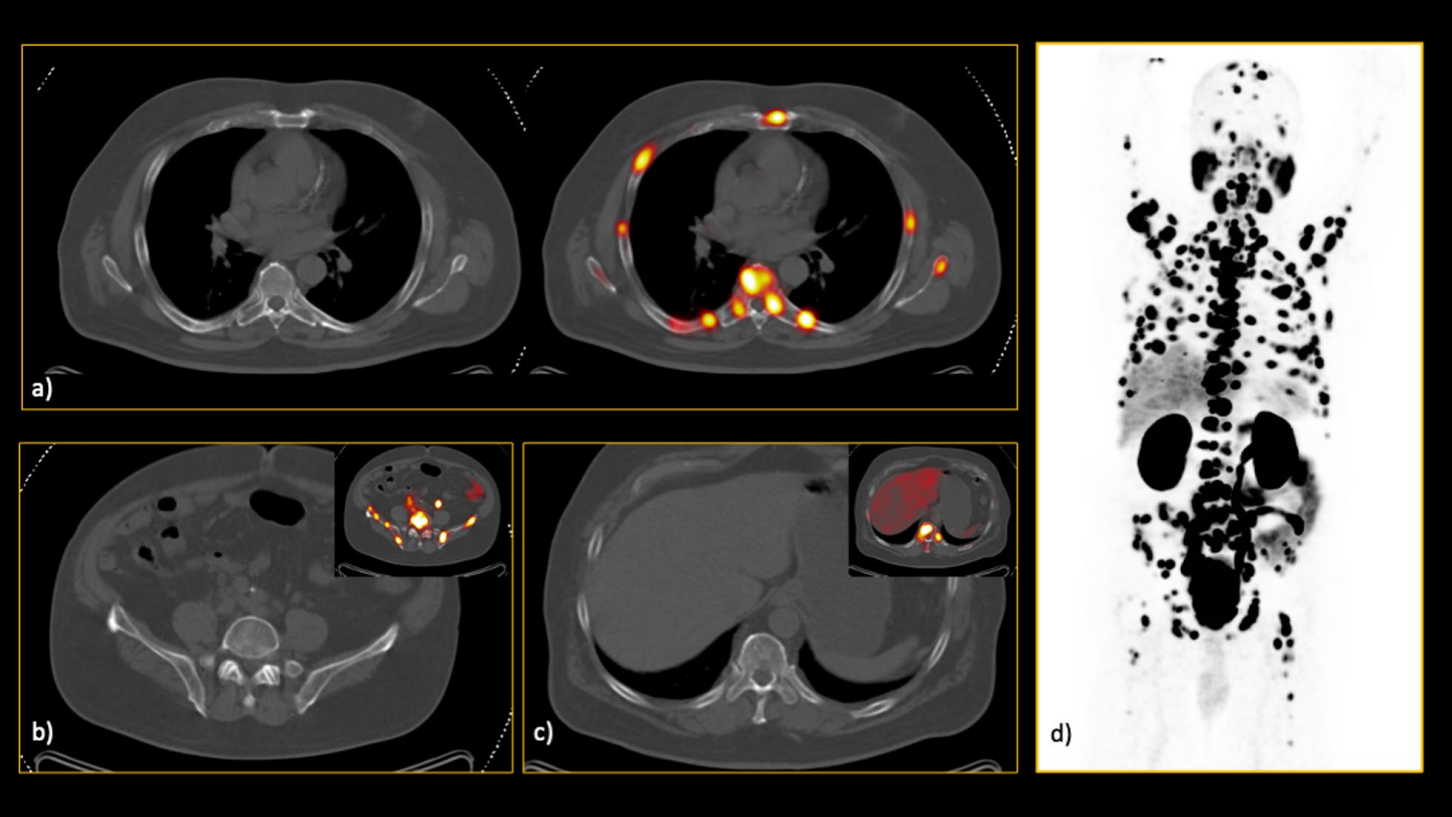
In this interesting case, a 66-year-old man referred for initial staging, there was extensive involvement of both axial and appendicular skeleton. However, none of the lesions had significant characteristics on the low-dose CT images, resulting in calling this patient free of skeletal involvement in CT evaluation by all readers. As you can see in the hybrid ^68^Ga-PSMA PET/CT images, there were innumerable bone metastases which all were invisible on CT alone (**a**–**c**). **d** The maximum intensity projection (MIP) clearly visualises the extent of the disease

Based on the reference standard modality, bone metastasis was present in 189/601 (31.4%) patients, of which 87 (46%) were extensive. In the extensive group, only one patient was falsely diagnosed as non-metastatic on CT images by all readers (Fig. 2). In this group, the lesions were found throughout the axial and appendicular skeleton, except in one patient that was confined to vertebrae and pelvic bones.

In the non-extensive group (*n* = 102), 10 (10%) patients had only lytic lesions, 16 (16%) had both lytic and sclerotic lesions, and 76 (75%) had only sclerotic lesions. The most common location of metastasis in this group was pelvic bones, seen in 81/102 (79.4%) patients, followed by vertebrae (55/102, 53.9%) and ribs (36/102, 35.3%). Regarding the missed lesions via CT images, the most common location was the pelvic bones (ranging from 25.2 to 31.1% among reviewers), followed by ribs (18.4–21.4%) and vertebrae (16.5–20.4%). Table 1 demonstrates characteristics of included patients.

**Table 1:** Characteristics of the included patients

	Initial staging (*n* = 211)	Biochemical recurrence (*n* = 390)
Age (years)	69.1 ± 8.7	68.5 ± 7.7
Bone metastasis	69 (32.7%)	120 (30.8%)
Extensive metastasis	31 (14.7%)	56 (14.6%)
Site of metastasis		
Pelvis	51	30
Spine	25	30
Rib	17	19
Humerus	2	6
Femur	9	10
Scapula	9	8
Skull	2	6
Sternum	2	4
Clavicle	0	2
Nature of metastasis		
Purely lytic metastasis	4	6
Mixed lytic-sclerotic metastasis	6	10
Purely sclerotic metastasis	28	48
Metastasis with fracture	2	9
Metastasis with soft-tissue component	3	7

### Per-patient analysis

Per-patient diagnostic analysis for three reviewers showed 81.0–89.4% sensitivity and 96.6–98.5% specificity for CT in detecting osteo-metastasis (Table 2a). In the staging group, 76.8–88.4% sensitivity and 98.6–99.3% specificity were calculated. The BCR group showed sensitivity and specificity of 83.3–90.0% and 95.6–98.6%, respectively. The senior radiologists had the highest sensitivity and specificity among the reviewers. Detailed calculated diagnostic performances are provided in the supplementary file.

**Table 2:** Diagnostic performances in the (a) per-patient and (b) per-lesion analyses

	Expertise	Sensitivity% (95% CI)	Specificity% (95% CI)	NPV% (95% CI)	PPV% (95% CI)	Accuracy% (95% CI)	TP	TN	FP	FN
*(a) Per-patient*										
All patients	Junior radiologist	83.6 (77.5–88.6)	96.6 (94.4–98.1)	92.8 (90.3–94.7)	91.9 (87.0–95.0)	92.5 (90.1–94.5)	158	398	14	31
	Nuclear medicine	81.0 (74.6–86.3)	98.5 (96.9–99.5)	91.9 (89.4–93.8)	96.2 (92.0–98.3)	93.0 (90.7–94.9)	153	406	6	36
	Senior radiologist	89.4 (84.1–93.4)	98.5 (96.9–99.5)	95.3 (93.1–96.9)	96.6 (92.7–98.4)	95.7 (93.7–97.2)	169	406	6	20
Patients referred for initial staging	Junior radiologist	78.3 (66.7–87.3)	98.6 (95.0–99.8)	90.3 (85.6–93.6)	96.4 (87.2–99.3)	91.9 (87.4–95.2)	54	140	2	15
	Nuclear medicine	76.8 (65.1–86.1)	98.6 (95.0–99.8)	89.7 (85.1–93.1)	96.4 (86.9–99.1)	91.5 (86.9–94.9)	53	140	2	16
	Senior radiologist	88.4 (78.4–94.9)	99.3 (96.1–100)	94.6 (90.2–97.1)	98.4 (89.6–99.8)	95.7 (92.1–98.0)	61	141	1	8
Patients referred after biochemical recurrence	Junior radiologist	86.7 (79.3–92.2)	95.6 (92.4–97.7)	94.2 (91.1–96.2)	89.7 (83.2–93.8)	92.8 (89.8–95.2)	104	258	12	16
	Nuclear medicine	83.3 (75.4–89.5)	98.5 (96.3–99.6)	93.0 (89.9–95.2)	96.2 (90.4–98.5)	93.9 (91.0–96.0)	100	266	4	20
	Senior radiologist	90.0 (83.2–94.7)	98.6 (95.7–99.4)	95.7 (92.7–97.4)	95.6 (90.1–98.1)	95.6 (93.1–97.4)	108	265	5	12
*(b) Per-lesion*										
All lesions	Junior radiologist (n = 518)	51.5 (45.7–57.2)	82.1 (76.1–87.2)	52.1 (48.8–55.4)	81.77 (76.5–88.1)	63.5 (59.1–67.7)	157	161	35	148
	Nuclear medicine (n = 427)	48.5 (42.8–54.3)	88.9 (81.0–94.3)	35.9 (33.0–39.0)	93.1 (88.4–96.0)	58.4 (53.4–63.3)	148	88	11	157
	Senior radiologist (n = 454)	63.3 (57.6–68.7)	92.1 (86.0–96.2)	51.1 (47.2–55.0)	95.1 (91.3–97.2)	71.8 (67.3–76.0)	193	117	10	112

*CI* confidence interval, *TP* true positive, *TN* true negative, *FP* false positive, *FN* false negative

### Per-lesion analysis

**Fig. 3:**
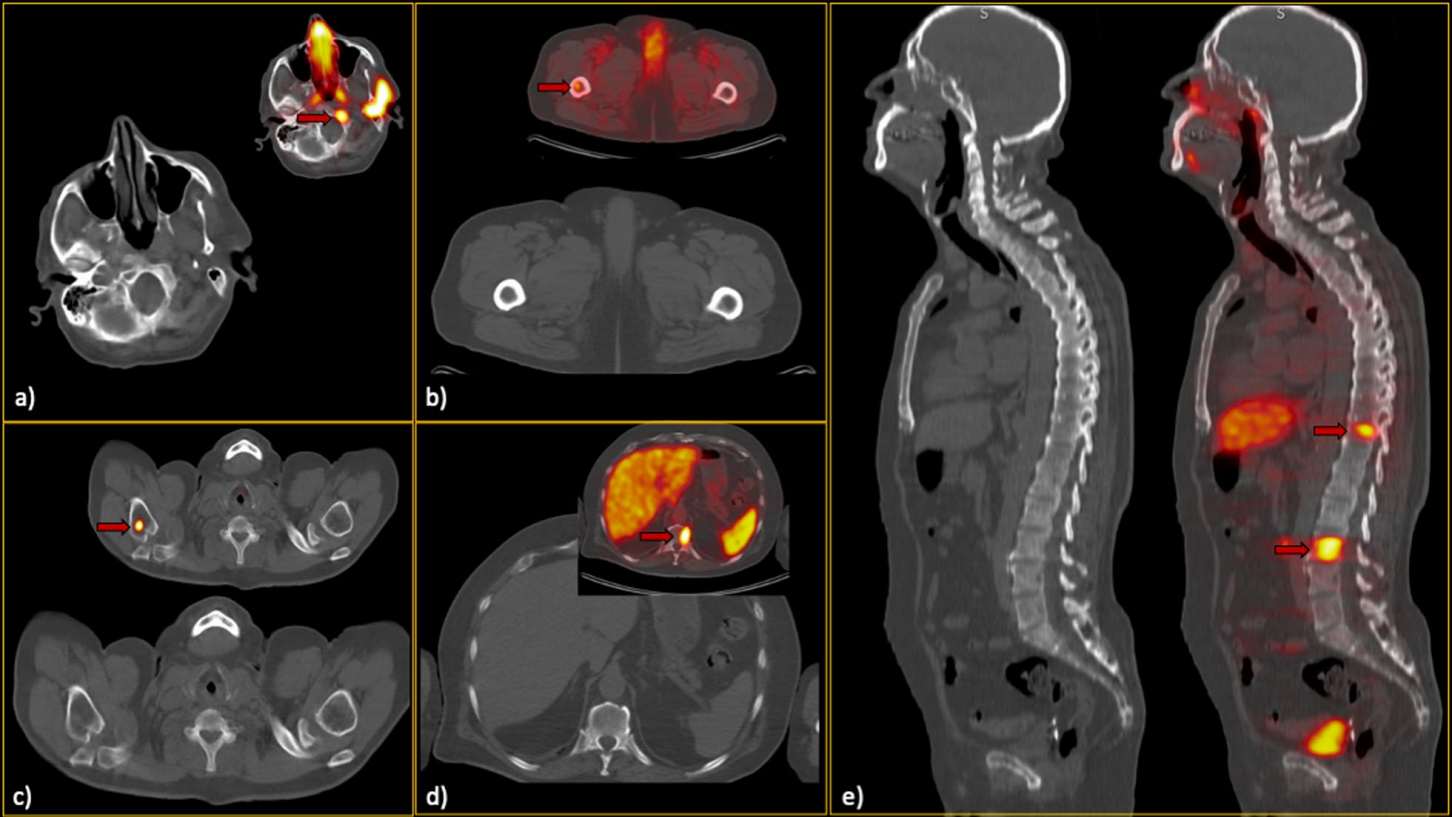
False-negative low-dose CT findings (red arrows). In each section (**a**–**e**), the CT-alone images, along with the hybrid ^68^Ga-PSMA PET/CT images, can be found. Regarding section a), it should be noted that he was one of our interesting cases, as he showed only a single bone metastasis in the skull base. Thus, although the sclerotic lesion can be easily seen in this single slice, it was not prominent enough while scrolling, since the remainder of the skeleton was free of metastases. Furthermore, in section c), subtle sclerosis could be found retrospectively; however, it was not considered a significant lesion by all readers when reviewing CT images. Notably, this false finding did not misclassify the patient’s disease burden eventually

**Fig. 4:**
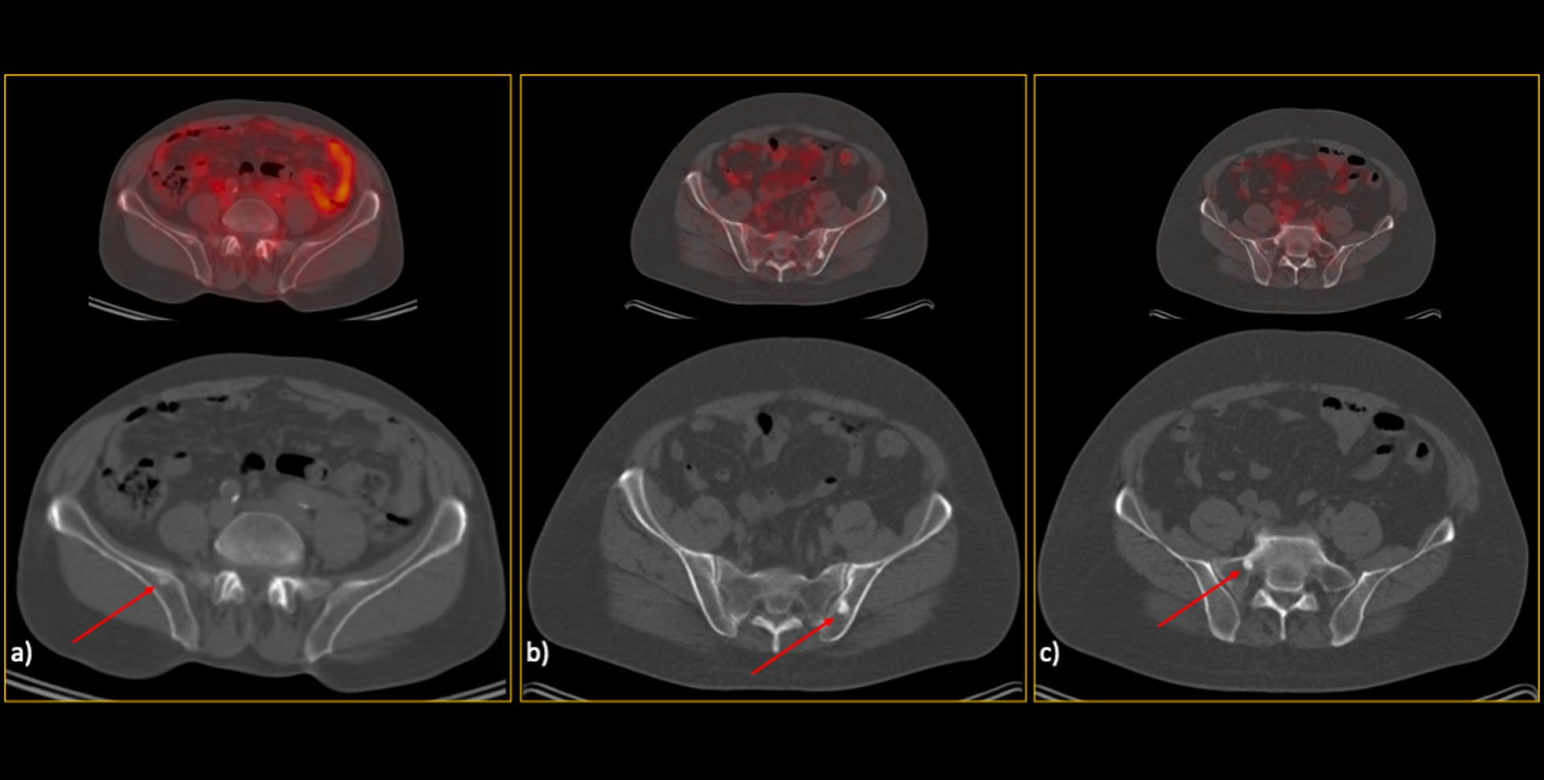
False-positive low-dose CT findings (red arrows). The majority of the false-positive findings were located in the pelvic region, most likely due to the readers’ knowledge about the high pretest probability in this region. So, as can be seen (**a**–**c**), there were various lesions called metastatic on CT images but were found benign considering the reference standard. Noteworthy, although **b** and **c** might seem not that much challenging and in favour of bone islands while looking at only one slice, in patients with multiple metastases, they were falsely interpreted as malignant since there were also other lesions with more or less similar Hounsfield unit in the same patient

**Fig. 5:**
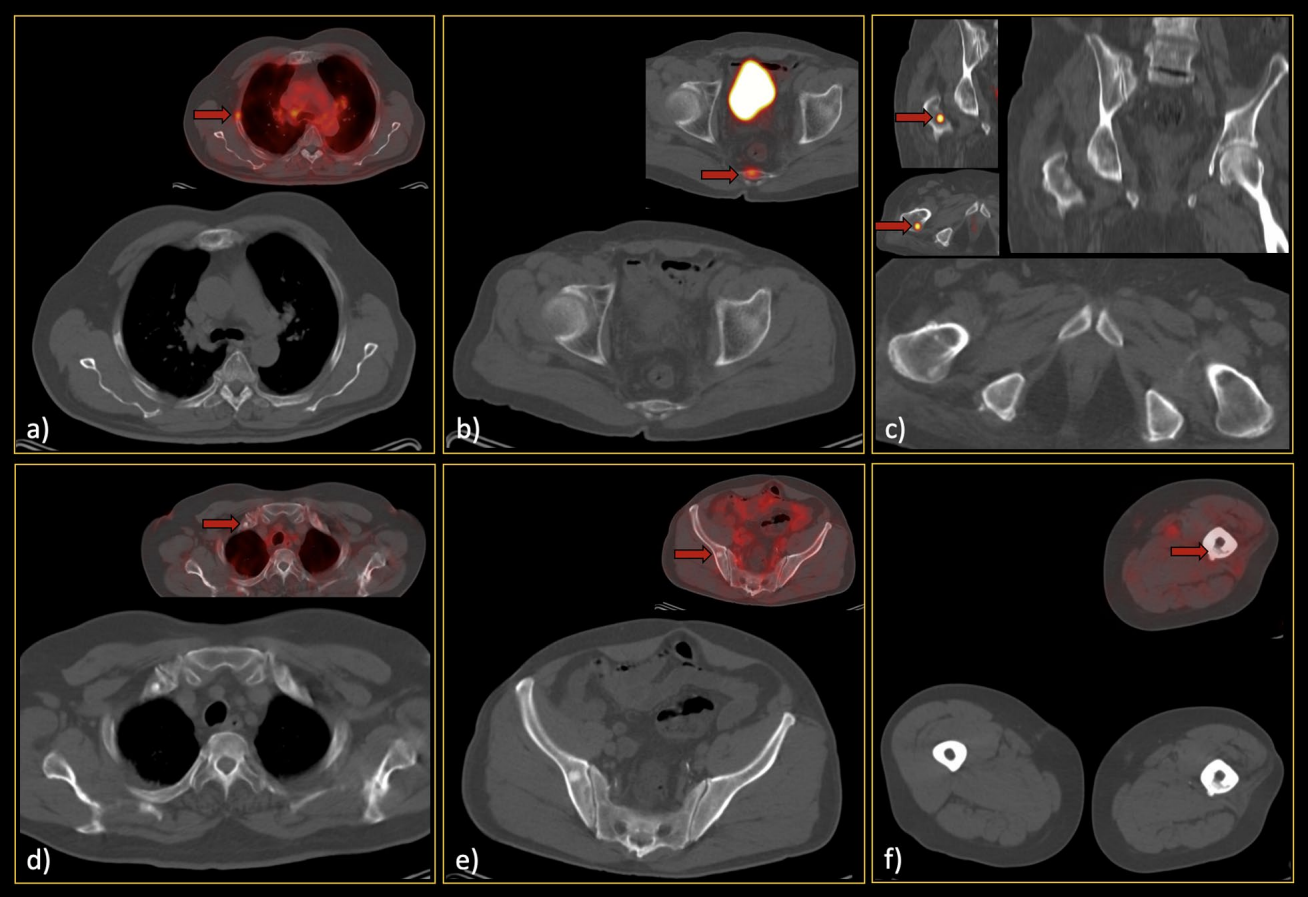
Lesions that were wrongly classified (**a**–**c** false negatives; **d**–**f** false positives) by one of the readers while correctly diagnosed by others. Thus, attention to these locations/kinds of lesions should be paid since they may also be missed on CT readings. Red arrows show the location of the metastases on the hybrid ^68^Ga-PSMA PET/CT images

In non-extensive patients, 305 metastatic lesions were found. The most common site was pelvic bones (127, 41.6%), followed by vertebrae (70, 23.0%) and ribs (42, 13.8%). Among all lesions, 94 (30.8%) metastases were not visible on CT images. Additionally, 204 benign lesions (based on the reference standard) were selected for comparative analyses. The detailed data of the selected lesions are provided in Table 2b. Per-lesion analysis showed sensitivity and specificity of 48.5–63.3% and 82.1–92.1% among reviewers, respectively. Figures 3 and 4 show representative lesions with false-negative and false-positive findings, respectively. Figure 5 depicts disagreements between readers.

**Fig. 6:**
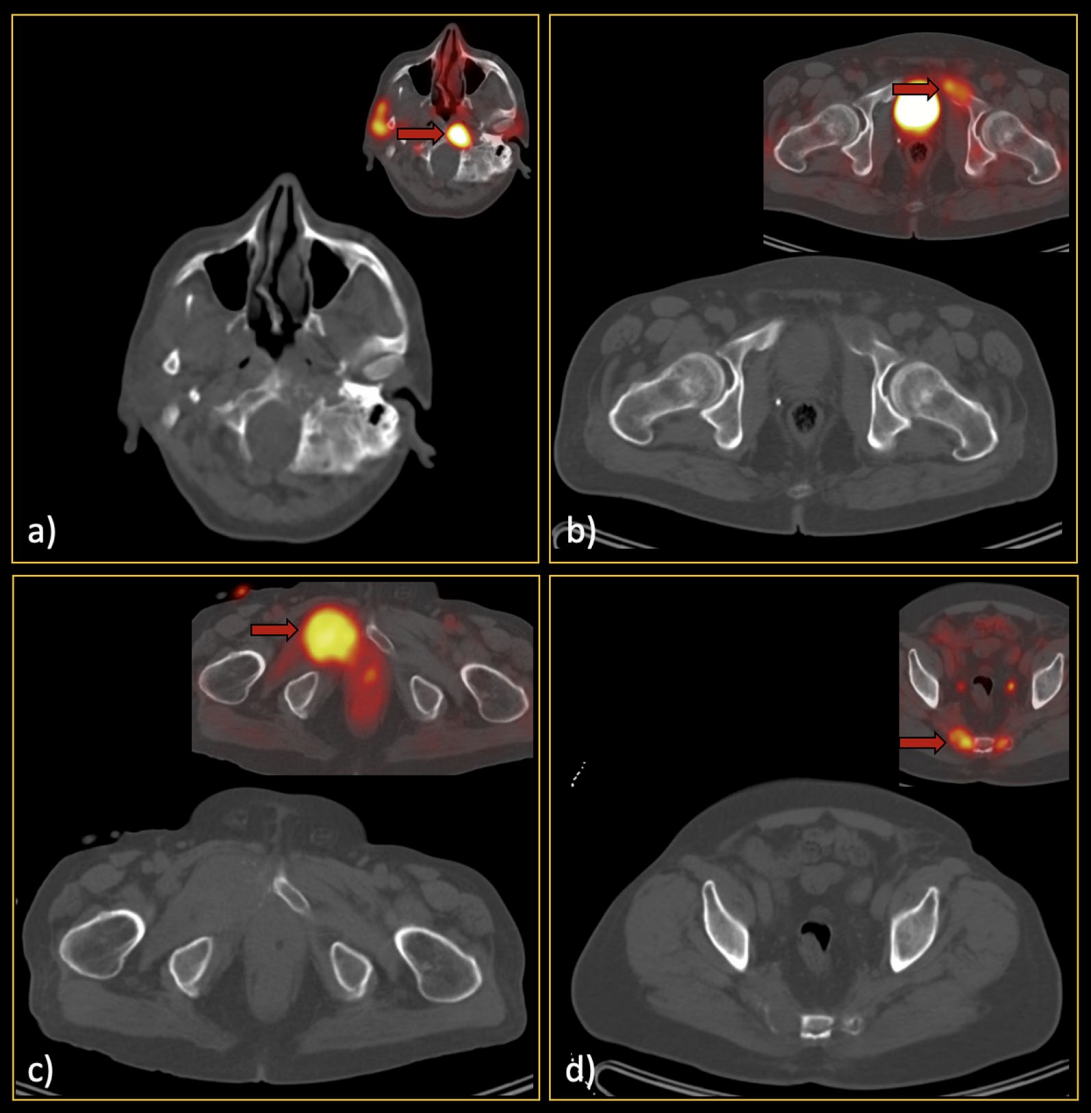
Examples of the typical bone metastases (**a**–**d**) with the detected prominent features, including soft tissue component, ill-defined border, cortical destruction, and maximal transverse diameter of more than one centimetre. Locations of the metastases are shown with red arrows on the hybrid ^68^Ga-PSMA PET/CT images

We also analysed all metastatic lesions with underlying CT findings to determine the features that could be useful in differentiating the benign and metastatic lesions (Table 3). Among the evaluated features, the analysis showed that size > 1 cm, ill-defined borders, presence of soft tissue component, and cortical destruction were statistically in favour of metastasis (Fig. 6). Also, Hu was significantly different among the two groups (*p* value < 0.001). The ROC curve analysis showed that lesions with more than 900 Hu were benign with 93% specificity.

**Table 3:** Characteristics of the included bone lesions

Characteristic of bone lesions	Metastatic (*n* = 305) number (%)	Benign (*n* = 204) number (%)	Odds ratio (95% CI)	*p* value
Site of detected lesion				
Pelvis	127 (41.6)	91 (44.6)	*NA*	*NA*
Vertebrae	70 (23.0)	57 (27.9)		
Rib	42 (13.8)	32 (15.6)		
Humerus	11 (3.6)	4 (2.0)		
Femur	20 (6.5)	12 (5.9)		
Scapula	19 (6.2)	4 (2.0)		
Skull	7 (2.3)	0 (0)		
Sternum	7 (2.3)	2 (1.0)		
Clavicle	2 (0.7)	2 (1.0)		
Nature				
Purely lytic	21 (6.9)	16 (7.8)	*NA*	0.696*
Purely sclerotic	174 (57.1)	175 (85.8)		
Mixed	16 (5.2)	13 (6.4)		
No underlying CT finding	94 (30.8)	–		
Soft tissue component	17 (5.6)	4 (2.0)	**3.0 (1.0–8.9)**	**0.044**
Ill-defined lesion	99 (32.5)	30 (14.7)	**2.8 (1.8–4.4)**	** < 0.001**
Size > 1 cm	161 (85.6)	68 (33.3)	**2.2 (1.5–3.2)**	** < 0.001**
Cortical destruction	24 (7.9)	7 (3.4)	**2.4 (1.0–5.7)**	**0.040**
Asymmetric growth	132 (43.3)	74 (36.3)	*NA*	0.114
Mean Hounsfield unit	537 ± 244.3	809.4 ± 376.5	*NA*	** < 0.001**

Bolded* p*-values are < 0.05

*p value was calculated among the lesions that had underlying CT finding

*NA not applicable

### Clinical practice-based per-patient analysis

To evaluate the diagnostic performance of CT in determining the treatment approach of the patients, separate analyses on both staging and BCR groups were performed. The detailed data are provided in Table 4. The results showed that CT had 88.6–92.4% accuracy among three reviewers when the CHAARTED criteria cut-off value was used. This meant that 7.6–11.4% of patients would not receive the appropriate treatment. Considering the LATITUDE criteria, the accuracy of the test was 88.2–93.4% among the three reviewers, showing that 6.6–11.8% of patients would receive inappropriate treatment. Table 4a demonstrates detailed information on this issue. The CT diagnostic performance in evaluating the osteo-metastasis in BCR patients (categorising them into non-metastatic, oligometastatic, and poly-metastatic groups) showed 90.3–94.4% accuracy.

**Table 4:** Diagnostic performance of CT based on the clinical significance (per-patient analysis)

Expertise	Sensitivity% (95% CI)	Specificity% (95% CI)	NPV% (95% CI)	PPV% (95% CI)	Accuracy% (95% CI)	TP	TN	FP	FN
*(a) Evaluation based on CHAARTED (Initial staging)*									
Junior radiologist	63.6 (49.6–76.2)	98.1 (94.5–99.6)	88.4 (84.4–91.6)	92.1 (78.9–97.3)	88.6 (83.6–92.6)	35	153	3	20
Nuclear medicine	60.3 (46.6–73.0)	98.7 (95.3–99.7)	86.8 (82.7–90.0)	94.6 (81.3–98.6)	87.7 (82.5–91.8)	35	151	2	23
Senior radiologist	71.2 (56.9–82.9)	99.4 (96.6–100)	91.3 (87.3–94.2)	97.4 (83.9–99.6)	92.4 (88.0–95.6)	37	158	1	15
*(b) Evaluation based on LATITUDE (Initial staging)*									
Junior radiologist	67.2 (53.7–79.0)	98.7 (95.4–99.8)	88.8 (85.0–92.0)	95.1 (83.0–98.7)	90.1 (85.2–93.7)	39	151	2	19
Nuclear medicine	62.3 (49.0–74.4)	98.7 (95.3–99.8)	86.6 (82.3–89.9)	95 (82.6–98.7)	88.2 (82.3–89.9)	38	148	2	23
Senior radiologist	76.4 (63.0–86.8)	99.4 (96.5–100)	92.3 (88.1–95.0)	97.7 (85.6–99.7)	93.4 (89.1–96.3)	42	155	1	13
*(c) Osteo-metastasis evaluation in biochemical recurrent patients*									
Junior radiologist	72.9 (62.9–81.5)	95.9 (93.0–97.9)	91.6 (88.6–93.8)	85.4 (78.8–91.1)	90.3 (86.9–93.0)	70	282	12	26
Nuclear medicine	70.4 (60.3–79.2)	98.6 (96.5–99.6)	90.9 (88.0–93.1)	94.5 (86.6–97.9)	91.5 (88.3–94.1)	69	288	4	29
Senior radiologist	81.3 (71.8–88.7)	98.3 (96.1–99.5)	94.4 (91.8–96.4)	93.7 (86.1–97.3)	94.4 (91.6–96.4)	74	294	5	17

CI confidence interval, TP true positive, TN true negative, FP, false positive, FN false negative

### Interobserver agreement

The per-lesion interobserver agreement among the three reviewers was almost perfect (91%). The highest inter-observer agreement was between the junior and senior radiologists (93.0%, 95% CI 90.1–95.9%), while the lowest agreement was between the junior radiologist and nuclear medicine specialist (88.3%, 95% CI 84.7–91.9%). Also, the agreement between the senior radiologist and nuclear medicine specialist was almost perfect (90.1%, 95% CI 86.6–93.5%).

## Discussion

In this study, we showed that despite the low diagnostic performance of CT in detecting all PCa bone metastatic lesions, the test provided accurate results in the clinical decision-making of the patients. The high accuracy at the per-patient level indicated that CT could correctly diagnose the presence or absence of metastasis in most patients. However, in clinical management, both in staging and BCR conditions, it is important to know the burden of disease. In this issue, CT still had reliable performance, meaning most patients would receive the proper therapy. Although the results of the three reviewers indicated the level of expertise importance in the diagnostic performance, the inter-observer analysis stated that the test could be confidently interpreted in decision-making.

Based on the latest guideline of PCa management, determining the burden of bone metastasis in initial staging is one of the factors that could separate low and high-risk patients, which have different management approaches [2]. This risk assessment, mainly done by CHAARTED or LATITUDE criteria, is not strictly influenced by the exact number/location of metastasis. Therefore, correct categorisation of the patients is the fundamental challenge. Moreover, a similar concept is applicable in patients with BCR. Furthermore, the present guidelines recommend the addition of bone health agents in patients with the presence of metastasis [2, 5]. Also, in patients with local symptoms or high pathologic fracture risk, local therapies like radiotherapy or surgery are recommended [16].

Considering all these issues, instead of focusing on the per-lesion performance of CT, which is undoubtedly low, we aimed to find the clinical impact. In the initial staging of PCa patients based on the above-mentioned criteria, whole-body low-dose CT showed promising results in terms of specificity and PPV, regardless of readers’ expertise. This means that the treatment approach would have been confidently adopted in the patients diagnosed with the high-burden disease. Having said that, the cost-effectiveness should be assessed to justify the NPV values.

Although using ^68^Ga-PSMA PET/CT is growing, BS is still widely used and recommended due to its accessibility and low price. In a recently published meta-analysis, the per-patient sensitivity and specificity of this modality were 86% (95% CI 76–92%) and 95% (95% CI 87–98%), respectively, which is similar to the performance of CT in our study [17]. In an older meta-analysis by Shen et al., they found a per-lesion sensitivity and specificity of 79% (95% CI 73–83%) and 82% (95% CI 78–85%), respectively, showing higher sensitivity compared to our results [18]. However, as mentioned earlier, finding all lesions may be not crucial in PCa management. Also, comparing CT and BS, CT is superior in providing anatomical details of the involved bones [19]. Thus, as osteo-metastasis local therapy is recommended in symptomatic patients or at high risk of pathologic fracture, CT could provide the essential information [20]. Besides, concurrent evaluation of the chest is also possible with the performed low-dose CT, showing a similar detection rate in the pulmonary nodules to the standard chest-CT [21, 22]. Also, the time spent for CT is much less than BS which could be important in patients’ compliance [19]. Noteworthy, although irradiation of CT could be high, the reported values for whole-body low-dose CT are comparable with BS [23–25].

Evaluation of metastatic lesions showed that the most common sites of metastasis were pelvic bones and vertebrae, which was concordant with previous studies [26]. Regarding the morphological texture, the evaluation of Hu revealed that lesions with > 900 Hu could be interpreted as benign with high specificity. Also, similar to previous studies, we found that the presence of soft-tissue component, ill-defined borders, cortical destruction, and > 1 cm lesions could be more in favour of metastasis [27]. Although most of these findings are not so common to see, it has been reported that adding morphologic CT features could even increase the diagnostic performance of ^68^Ga-PSMA PET [8].

There are some limitations in this study. First, we did not provide readers with clinical and lab data of the patients, particularly the serum PSA level, while reading scans. This could be a double-edged sword, since although we might not assess the readers’ performance in the real clinical routine, we solely relied on the knowledge of readers in terms of imaging to purposefully take CT-alone potential into account. Second, since histopathology exam was not widely available in bone lesions, we used ^68^Ga-PSMA PET/CT, which, albeit not perfect, has been shown to be highly accurate in bone assessment. Thus, although rare false-positive lesions (e.g. hemangioma, Paget’s disease) and equivocal cases were excluded by nuclear medicine specialists (additional imaging results or patient follow-up in challenging cases were sought) or eventually from the analyses when remained equivocal, a few false-negative findings (e.g. purely sclerotic lesions) could still be misdiagnosed by our reference standard [28]. Third, we were faced with referral bias. Since our centre was the centre of excellence for ^68^Ga-PSMA PET/CT in the country, it was possible that many complicated or misdiagnosed patients would be referred to us. This could potentially lead to the underestimation of NPV results in a population with a high pretest probability. Lastly, the retrospective nature of the study had its own limitations, and the real impact of the findings on patient management could not be truly analysed.

In conclusion, we found that whole-body low-dose CT could provide reliable data in the skeletal assessment needed for PCa patient management. Of course, ^68^Ga-PSMA PET/CT has much better performance in evaluating locoregional and distant metastasis and could not be replaced by CT; however, the performance of CT in the skeleton could be comparable with BS. Additionally, we proposed some diagnostic CT features that could help radiologists with better characterisation of the lesions. Thus, simple and available imaging like CT with the reported high specificity and PPV may significantly help decision-making in PCa patients, reserving the high-cost modalities in cases with negative results on the CT images.

## Data Availability

The detailed data generated during and/or analysed during the current study are available from the corresponding author upon reasonable request.

## Abbreviations

BCR: Biochemical recurrence
BS: Bone scintigraphy
CT: Computed tomography
Hu: Hounsfield unit
NPV: Negative predictive value
PCa: Prostate cancer
PET: Positron emission tomography
PPV: Positive predictive value
ROC: Receiver operating characteristic

## References

[1] Azad GK, Taylor B, Rubello D, Colletti PM, Goh V, Cook GJ (2016). Molecular and functional imaging of bone metastases in breast and prostate cancers: an overview. Clin Nuc Med.

[2] Parker C, Gillessen S, Heidenreich A, Horwich A (2020). Prostate cancer: ESMO Clinical Practice Guidelines for diagnosis, treatment and follow-up. Ann Oncol.

[3] Sweeney CJ, Chen YH, Carducci M (2015). Chemohormonal therapy in metastatic hormone-sensitive prostate cancer. N Engl J Med.

[4] Fizazi K, Tran N, Fein L (2017). Abiraterone plus prednisone in metastatic, castration-sensitive prostate cancer. N Engl J Med.

[5] Schaeffer E, Srinivas S, Antonarakis ES (2021). Prostate cancer, version 1.2021: Featured updates to the nccn guidelines. J Natl Compr Can Netw.

[6] Esen T, Kılıç M, Seymen H, Acar Ö, Demirkol MO (2020). Can Ga-68 PSMA PET/CT replace conventional imaging modalities for primary lymph node and bone staging of prostate cancer?. Eur Urol Focus.

[7] Sartor O, de Bono JS (2018). Metastatic prostate cancer. N Engl J Med.

[8] Janssen JC, Meißner S, Woythal N (2018). Comparison of hybrid 68Ga-PSMA-PET/CT and 99mTc-DPD-SPECT/CT for the detection of bone metastases in prostate cancer patients: additional value of morphologic information from low dose CT. Eur Radiol.

[9] Lengana T, Lawal IO, Boshomane TG (2018). 68Ga-PSMA PET/CT replacing bone scan in the initial staging of skeletal metastasis in prostate cancer: a fait accompli?. Clin Genitourin Cancer.

[10] Chavoshi M, Mirshahvalad SA, Metser U, Veit-Haibach P (2021). 68Ga-PSMA PET in prostate cancer: a systematic review and meta-analysis of the observer agreement. Eur J Nucl Med Mol Imaging.

[11] Heidenreich A, Bastian PJ, Bellmunt J (2014). EAU guidelines on prostate cancer. Part 1: screening, diagnosis, and local treatment with curative intent—update 2013. Eur Urol.

[12] Hövels A, Heesakkers RA, Adang EM (2008). The diagnostic accuracy of CT and MRI in the staging of pelvic lymph nodes in patients with prostate cancer: a meta-analysis. Clin Radiol.

[13] Kane CJ, Mitchell JA, Meng MV, Anast J, Carroll PR, Stoller ML (2003). Limited value of bone scintigraphy and computed tomography in assessing biochemical failure after radical prostatectomy. Urology.

[14] Mohler JL, Antonarakis ES, Armstrong AJ (2019). Prostate cancer, version 2.2019, NCCN clinical practice guidelines in oncology. J Natl Compr Can Netw.

[15] Jadvar H, Abreu AL, Ballas LK, Quinn DI (2022). Oligometastatic Prostate Cancer: Current Status and Future Challenges. J Nucl Med.

[16] Nguyen QN, Chun SG, Chow E (2019). Single-fraction stereotactic vs conventional multifraction radiotherapy for pain relief in patients with predominantly nonspine bone metastases: a randomized phase 2 component of a phase 2/3 trial. JAMA oncol.

[17] Zhou J, Gou Z, Wu R, Yuan Y, Yu G, Zhao Y (2019). Comparison of PSMA-PET/CT, choline-PET/CT, NaF-PET/CT, MRI, and bone scintigraphy in the diagnosis of bone metastases in patients with prostate cancer: a systematic review and meta-analysis. Skeletal Radiol.

[18] Shen G, Deng H, Hu S, Jia Z (2014). Comparison of choline-PET/CT, MRI, SPECT, and bone scintigraphy in the diagnosis of bone metastases in patients with prostate cancer: a meta-analysis. Skeletal Radiol.

[19] Langsteger W, Rezaee A, Pirich C, Beheshti M (2016) 18F-NaF-PET/CT and 99mTc-MDP bone scintigraphy in the detection of bone metastases in prostate cancer. In: Seminars in nuclear medicine 2016. Elsevier. 10.1053/j.semnuclmed.2016.07.003 10.1053/j.semnuclmed.2016.07.003 27825429

[20] Confavreux CB, Follet H, Mitton D, Pialat JB, Clezardin P (2021). Fracture risk evaluation of bone metastases: a burning issue. Cancers.

[21] Karabulut N, Törü M, Gelebek V, Gülsün M, Ariyürek MO (2002). Comparison of low-dose and standard-dose helical CT in the evaluation of pulmonary nodules. Eur Radiol.

[22] Wormanns D, Ludwig K, Beyer F, Heindel W, Diederich S (2005). Detection of pulmonary nodules at multirow-detector CT: effectiveness of double reading to improve sensitivity at standard-dose and low-dose chest CT. Eur Radiol.

[23] Lambert L, Ourednicek P, Meckova Z, Gavelli G, Straub J, Spicka I (2017). Whole-body low-dose computed tomography in multiple myeloma staging: Superior diagnostic performance in the detection of bone lesions, vertebral compression fractures, rib fractures and extraskeletal findings compared to radiography with similar radiation exposure. Oncol Lett.

[24] Leide-Svegborn S (2010). Radiation exposure of patients and personnel from a PET/CT procedure with 18F-FDG. Radiat Prot Dosimetry.

[25] Van den Wyngaert T, Strobel K, Kampen WU (2016). The EANM practice guidelines for bone scintigraphy. Eur J Nucl Med Mol Imaging.

[26] Dennis ER, Jia X, Mezheritskiy IS (2012). Bone scan index: a quantitative treatment response biomarker for castration-resistant metastatic prostate cancer. J Clin Oncol.

[27] Coleman RE, Brown J, Holen I (2020) Bone metastases. In: Abeloff's clinical oncology, 2020, pp. 809–830. e3. 10.1016/B978-0-323-47674-4.00056-6

[28] Uprimny C, Svirydenka A, Fritz J (2018). Comparison of [68Ga] Ga-PSMA-11 PET/CT with [18F] NaF PET/CT in the evaluation of bone metastases in metastatic prostate cancer patients prior to radionuclide therapy. Eur J Nucl Med Mol Imaging.

